# *Mycobacterium abscessus* pulmonary disease presenting with spontaneous pneumomediastinum and subcutaneous emphysema in childhood acute lymphoblastic leukemia: a case report and literature review

**DOI:** 10.1186/s12887-023-04199-4

**Published:** 2023-08-28

**Authors:** Wenyuan Liu, Jinhua Chu, Zhiwei Xie, Linhai Yang, Lingling Huang, Songji Tu, Huaju Cai, Zhengyu Wu, Anbang Wei, Chengzhu Liu, Yan Cheng, Kunlong Zhang, Ningling Wang

**Affiliations:** Department of Pediatrics, The Second Affiliated Hospital of Anhui Medical University, Anhui Province, No. 678 Furong Road, Hefei City, 230601 China

**Keywords:** *Mycobacterium abscessus*, Pneumonia, Children, Acute lymphoblastic leukemia, Spontaneous pneumomediastinum, Subcutaneous emphysema

## Abstract

**Introduction:**

*Mycobacterium abscessus* is a rapidly growing mycobacterium commonly identified in adults with underlying pulmonary diseases but is rarely observed in children. A better understanding of this pathogen in children is essential.

**Case presentation:**

We report the case of a 49-month-old female child without previous underlying pulmonary diseases but with acute lymphoblastic leukemia (ALL). The patient was complicated with pneumonia during chemotherapy, which was primarily characterized by spontaneous pneumomediastinum and subcutaneous emphysema on chest computed tomography (CT). *M. abscessus* sequences were detected by metagenomic next-generation sequencing in bronchoalveolar lavage fluid. With mechanical ventilation, closed thoracic drainage, and anti-infective therapy for 6 months, the patient’s infection was controlled. The patient completed 2.5 years of treatment for ALL, and the drugs were discontinued. The patient currently remains in complete hematologic remission.

**Discussion:**

We reviewed the literature on 33 children with *M. abscessus* pulmonary disease. These children mostly had underlying immunodeficiency. Chest CT most often showed nodular shadows, consolidation, and bronchiectasis. Spontaneous pneumomediastinum and subcutaneous emphysema were not reported as major manifestations.

**Conclusion:**

Spontaneous pneumomediastinum and subcutaneous emphysema were our patient's main characteristics on chest CT, and this study enriches the knowledge regarding possible imaging changes in *M. abscessus* pulmonary disease in children. This case report reflects good clinical experience in maintaining the balance between chemotherapy and anti-infective therapy in childhood ALL.

**Supplementary Information:**

The online version contains supplementary material available at 10.1186/s12887-023-04199-4.

## Introduction

Acute lymphoblastic leukemia (ALL) is the most common malignancy in childhood globally, and the cure rate has been constantly increasing with technological advancements and medical science improvements [1, 2]. As with other malignancies, immunodeficiency leads to enhanced susceptibility to infection. *Mycobacterium abscessus* is a rapidly growing nontuberculous mycobacterium (NTM) that is ubiquitous in the environment, including in water, soil, and dust [3]. Post surgery/post trauma skin and soft tissue infection, lymphadenitis, pulmonary infection (usually with underlying pulmonary chronic diseases, such as cystic fibrosis), and disseminated infection (usually associated with immunodeficiency) are common childhood infections caused by *M. abscessus* [4]. Reports on childhood ALL complicated with *M. abscessus* infection are limited; thus, a better understanding of this pathogen in children is essential.

We describe a child with ALL but without previous underlying pulmonary diseases. The patient developed *M. abscessus* pulmonary disease during chemotherapy, which was primarily characterized by spontaneous pneumomediastinum and subcutaneous emphysema. Her pulmonary infection was cured by a combination of antibiotics that were taken for 6 months after diagnosis, and she is still in complete hematologic remission from ALL at 2.5 years after the initial diagnosis.

## Case presentation

A 49-month-old female child was admitted to our hospital in December 2019 due to pallor for the prior 3 months. She had a normal growth and development history. She was a full-term normal delivery. She had no family history of hematological malignancies. She had suffered from pallor 3 months prior, which was treated using oral iron supplementation, but her symptoms did not improve and were accompanied by fatigue and anorexia. She had experienced intermittent low-grade fevers during the previous month and gingival bleeding 3 days prior and therefore visited a local hospital. Blood tests indicated a white blood cell count of 75.3×10^9^/L(3.5-9.5×10^9^/L), a hemoglobin level of 41 g/L (115-150g/L) and a platelet count of 14×10^9^/L(125-350×10^9^/L). She was then transferred to our department for further diagnosis and treatment.

Physical examinations on admission showed an anemic appearance; scattered bleeding spots on the skin over the entire body; palpable enlarged lymph nodes in the neck, armpit, and groin; the liver was located 4 cm below the ribs, which had a moderate texture; and the spleen was located 7 cm below the ribs, which had a tough texture. Routine blood tests indicated a white blood cell count of 67.19×10^9^/L(3.5-9.5×10^9^/L), a lymphocyte ratio of 89.4% (20-50%), a hemoglobin level of 41 g/L (115-150g/L), and a platelet count of 19×10^9^/L(125-350×10^9^/L). LDH was 577U/L(120-250U/L), HS-CRP was 7.1mg/L(0-3.3mg/L), and PCT was normal. Abdominal B-ultrasound revealed hepatosplenomegaly. No obvious abnormalities were found on head MRI, lung computed tomography (CT), electrocardiogram, color Doppler echocardiography, or urinary B-ultrasound. The patient was diagnosed with B-cell ALL (B-ALL) based on her bone marrow cell morphology, immunology, cytogenetics, and molecular biology (MICM) typing. The Chinese Children’s Cancer Group study ALL-2015 (CCCG-ALL-2015) intermediate-risk group regimen was initiated.

**Fig. 1:**
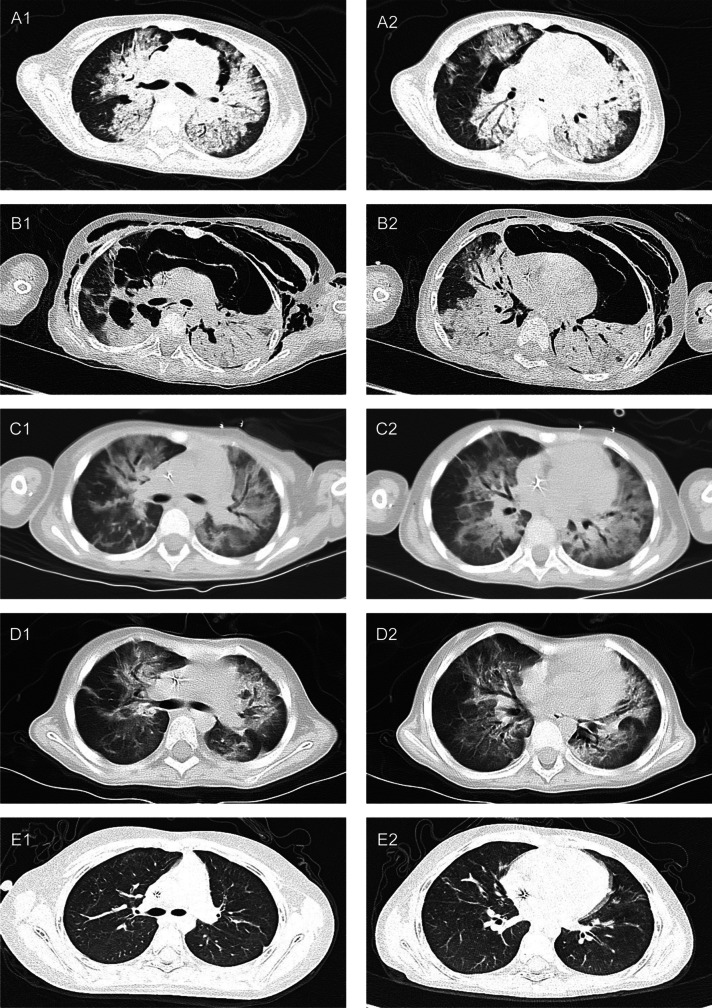
Chest CT results during the course of the disease. Chest CT performed on January 7, 2020(VDLP D23) (A1, A2), on January 8, 2020(VDLP D24) (B1, B2), on January 17, 2020(VDLP D33) (C1, C2),on February 1, 2020(D1, D2), and on June 28, 2020(E1, E2)

On day 8 of vincristine + daunorubicin + L-asparaginase + prednisone (VDLP) chemotherapy (December 23, 2019), the patient developed fever, cough, and moist rales in her lungs. Sputum bacterial and fungal cultures, bilateral blood cultures, and tuberculosis tests were negative. Empirically, she was intravenously injected with cefoperazone sulbactam (160 mg/kg/d) and orally given posaconazole (12 mg/kg/d) and linezolid (30 mg/kg/d). On day 18 of VDLP chemotherapy (January 2, 2020), she developed another fever, abdominal distension, abdominal pain, and weak bowel sounds, and a vertical abdominal X-ray indicated an intestinal obstruction. Sputum culture and blood culture were still negative, and a stool culture was normal. The anti-infective regimen was switched to intravenous injection of imipenem and cilastatin sodium (60 mg/kg/d), linezolid (30 mg/kg/d), and micafungin (4 mg/kg/d). She was also prescribed fasting gastrointestinal decompression and maintenance of water and electrolyte balances. On day 23 of VDLP chemotherapy (January 7, 2020), she suddenly had shortness of breath. Arterial blood gas analysis revealed a pH of 7.475, a partial pressure of oxygen (PO_2_) of 40.2 mmHg, a partial pressure of carbon dioxide (PCO_2_) of 38.5 mmHg, and a ratio of arterial oxygen pressure (PaO_2_)/fraction of inspired oxygen (FiO_2_) of 135 mmHg. A plain CT scan showed bilateral lung inflammation, partial consolidation and pneumomediastinum (Fig. 1-A1 and A2). She was endotracheally intubated for invasive mechanical ventilation (VC-AC mode) with the following parameters: FiO_2_ 40%, peak inspiratory pressure 24 cmH_2_O, positive end-expiratory pressure 5 cmH_2_O, respiratory rate 25/minute, and inspiratory time 0.8 s. She also underwent fiberoptic bronchoscopy. Bronchoalveolar lavage fluid (BALF) was collected and sent for metagenomic next-generation sequencing (mNGS), which was performed by BGI (Shenzhen, China) using the BGISEQ-500 (China). BALF was also sent for bacterial culture, fungal culture, antacid staining, ink staining, hexamine silver staining, and a galactomannan (GM) test, and the results were negative. On day 24 of VDLP chemotherapy (January 8, 2020), a new chest CT scan showed subcutaneous emphysema, pneumomediastinum, and atelectatic lung tissues under compression (Fig. 1-B1 and B2). Closed thoracic drainage and subcutaneous emphysema cutting for decompression were performed. mNGS detected *M. abscessus* with 515 sequence reads and *Mycobacteroides franklin* with 4 sequence reads in the BALF sample (the sequencing files were deposited into the NCBI SRA database and can be retrieved at https://www.ncbi.nlm.nih.gov/ with accession number PRJNA882796). The patient was additionally given intravenous azithromycin (10 mg/kg/day, 3 days a week). On day 30 of VDLP chemotherapy (January 14, 2020), invasive mechanical ventilation was discontinued. On day 33 of VDLP chemotherapy (January 17, 2020), a new chest CT scan revealed that both pneumatosis and inflammation had improved (Fig. 1-C1 and C2), and the patient was transferred back to the general ward for continued treatment.

**Fig. 2:**
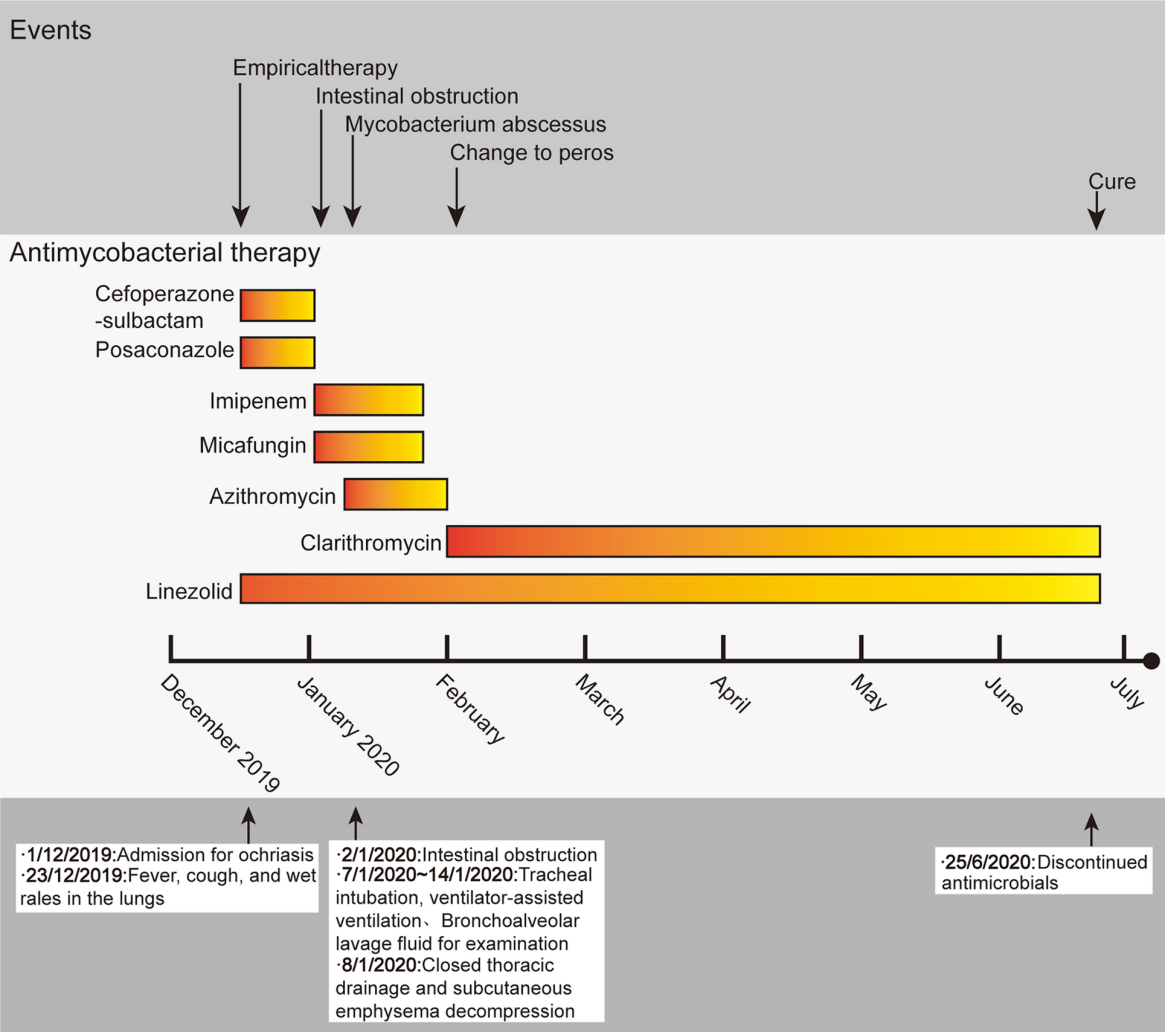
Timeline of events in the case

On January 24, 2020, the patient’s routine blood test, C-reactive protein, and procalcitonin results were all normal; therefore, cyclophosphamide + cytarabine + mercaptopurine (CAT) chemotherapy was started, and the anti-infective regimen was switched to oral linezolid (30 mg/kg/d) and clarithromycin (15 mg/kg/d) (Fig. 2). On day 7 of CAT (February 1, 2020), chest CT revealed that inflammation had been absorbed and had subsided more than before (Fig. 1-D1 and D2). At around 4 (June 28, 2020) of interphase treatment (mercaptopurine + dexamethasone + daunorubicin + vincristine + pegaspargase), chest CT revealed that the inflammation in both lungs had been absorbed (Fig. 1-E1 and E2). Therefore, the anti-infective therapy that had been given for 6 months was discontinued (Fig. 2). Chemotherapy was continued for ALL.

Fortunately, the 2.5-year chemotherapy course was completed in June 2022, and the patient currently remains in hematologic remission. During this period, the patient has had no symptoms such as cough and fever.

## Discussion

*M. abscessus* is a rapidly growing NTM of Runyon group IV that is ubiquitous in nature [5]. It is usually nonpathogenic in immunocompetent populations but often attacks immunodeficient, trauma, and postsurgical patients as an opportunistic pathogen [6, 7]. Organ infections, such as pulmonary infection [8], lymphadenitis [9], and disseminated infection [10], can be caused by *M. abscessus*. According to a recent whole-genome sequencing analysis of *M. abscessus* strains in patients with pulmonary cystic fibrosis, *M. abscessus* strains have high homology, suggesting that *M. abscessus* can be transmitted between people [11].

Searching PubMed with the keywords “*Mycobacterium abscessus*” and “Children” from January 2012 to June 2022 yielded a total of 195 articles. We applied the following inclusion criteria to the papers: 1) studies published in English, including case reports and case series, 2) studies involving pulmonary infection caused by *M. abscessus* in patients aged below 18 years, and 3) studies with clear basic information. Eighteen articles were included (Table 1). Pulmonary infection caused by *M. abscessus* was more common in patients with underlying pulmonary diseases, especially cystic fibrosis. In these cases, nodular shadows, consolidation, and bronchiectasis were the major CT manifestations of pulmonary infection in children. The primary characteristics observed in the lung CT of the present case encompassed bilateral pulmonary inflammation, pneumomediastinum, and subcutaneous emphysema. Spontaneous pneumomediastinum refers to mediastinal emphysema that occurs in circumstances other than exogenous trauma or iatrogenic injury [29]. The patient’s pneumomediastinum was found on chest CT before mechanical ventilation was initiated, which excludes the possibility of ventilator-associated mediastinal emphysema. We consider that it is related to lung infection. Spontaneous pneumomediastinum in patients with pneumonia has been reported in both adults and children [30, 31], but spontaneous pneumomediastinum in patients with *Mycobacterium abscessus* pulmonary disease is first reported in children. This is the first report in a pediatric case. The sudden worsening of respiratory symptoms and the decrease in PO2 caused by M. abscessus pulmonary disease indicate an exacerbation of pulmonary lesions. The patient’s lung CT scan revealed the presence of pneumomediastinum, which further enriches knowledge about the imaging presentation of *M. abscessus* pulmonary infections in children.

**Table 1:** Summary of mycobacterium abscessus complex lung infections

	**Age** **(years)**	**Sex**	**Medical history**	**Type of infection**	**Antibiotics**	**Total duration of treatment**	**Iconography**	**Outcome**
Do et al. (2013) [12]	8	-	CF	Lung	RIF,CFX,AMK,EMB,CAM,CIP	12 months	CT: Bronchiectasis with centrilobular nodularities, no tree-in-bud.	Failure
	13	-	Primary ciliary dyskinesia	Lung	AMK, MER, AZM	12 months	CT:RLL bronchiectasis with scattered tiny nodular densities, no tree-in-bud.	Cure
	0.5	-	No prior history	Lung	CFX,AMK, MER, CAM	3 months	CT: Extensive right lung consolidation with scattered nodular densities, no tree-in-bud.	Cure
Iwanaga et al, (2014) [13]	4	F	Bronchopulmonary dysplasia	Lung	CAM, AMK, LZD, TGC	13 months	CT: Chest demonstrated diffuse nodular opacities and areas of Consolidation	Cure
Jamal et al, (2014) [14]	1.33	-	CHD, Hypothyroidism, Chronic lung disease	Lung	AMK, CIP, CAM	-	New chest radiograph infiltrate	Cure
	12	-	HCE, Spastic quadriplegia,Recurrent chest infections, Chronic lung disease	Lung	AMK, CIP, CAM	-	New chest radiograph infiltrate	Cure
	1	-	CCHD, Congenital cystic lung,RLL lobectomy,Chronic lung disease	Lung	AMK, CIP, CAM	-	New chest radiograph infiltrate	Cure
	15	-	Spinomuscular dystrophy, Trachea-esophageal fistula, Scoliosis	Lung	AMK, CIP, CAM	-	New chest radiograph infiltrate	Failure
Apiwattankul et al,(2015) [15]	9	F	RMS	Blood, Lung	CAM, AMK,	6 months	-	Cure
	13	F	WT	Lung	CAM, LZD, AMK, MER	42 months	-	Failure
Iroh Tam et al,(2015) [16]	6	M	CF	Lung	Not treated	-	-	-
	12	M	CF	Lung	LVFX,TOB	-	-	-
	16	F	CF	Lung	Not treated	-	-	-
Emiralioğlu et al,(2016) [17]	11	M	Triple A Syndrome	Lung	IPM,AMK,CAM, DOX,CIP	24 months	X-RAY:Bilateral extensive nodular infiltrates, which were coalescing to form an area of consolidation in both lungs; CT:Multiple pulmonary nodules and consolidation in both lungs and an appearance of tree-in-bud.	Cure
Campos-Herrero et al, (2016) [18]	9	F	CF	Lung	CAM,AMK,LZD,TGC,MER,MOC,MXF	11 months	-	Failure
	9	F	CF	Lung		7 months	-	Improvement
	12	M	CF	Lung		41 months	-	Failure
	5	F	CF	Lung	Not treated	-	-	-
	11	M	CF	Lung		-	-	-
	12	F	CF	Lung		-	-	-
Anisowicz et al,(2016) [19]	8	F	Idiopathic short stature,allergic rhinitis,asthma	Lung	TGC,AZM,TOB,AMX,CVA,TZP,CLI	5 months	CT:Multifocal tree-in-bud opacities with associated diffuse bronchiectasis and ground glass opacities, wedgeshaped process in the right middle lobe concerning	Cure
Scott et al, (2018) [20]	10	F	CF	Lung	AMK,CFX,LZD	3.5 years	-	Cure
Ruffles et al, (2018) [21]	16	M	CF	Lung	AZM,EMB,MXF, AMK	7 months	CT: Progressive bronchiectasis	Cure
Liu et al, (2019) [22]	0.33	F	Born prematurely	Lung	CAM,IPM,LZD,RIF,EMB,CFX,AZM,MXF,CZ	16 months	X-RAY: Patchy shadows localized to the right lung and lower left lung; CT:Multiple masses and small nodules across both lungs with mediastinal lymph node involvement	Cure
Jones et al, (2019) [23]	16	F	CF	Lung	CFX,LZD,CAM,TGC,AMK,IMP/CS,MOC,TED	At least 5.5 years		Improvement
	13	M	CF	Lung	AMK,CFX,IMP/CS,LZD,MOC,TED,AZM	At least 5.6 years		Improvement
Mauch et al, (2020) [24]	13.3	F	Tracheobronchitis,CF	Lung	AMK,CAM	3 months	-	Cure
	15.8	M	Tracheobronchitis,CF	Lung	Not treated	-	-	Improvement
Alramadhan et al,(2021) [25]	0.33	F	Failure to thrive	Lung	AMK,CAM,IPM	14 months	-	Cure
Deniz et al, (2021) [26]	11	M	Autism, Pneumonia	Lung	LZD,AMK,CAM,RIF,INH,TMP-SMX	2 years	X-RAY: Lobar consolidation and interstitial infiltrates in the right lung; CT: Showed large, consolidated infiltration areas containing air bronchograms in the upper and middle lobes of the right lung and diffuse ground-glass opacities in the both lower lobes	Cure
Chawla et al, (2022) [27]	7	F	CF ,Failure to thrive, Meconium ileus,Volvulus	Lung	CLOF,LZD,CFX, AMK,TGC	Estimate 24 months	X-RAY:Left lower lung focal consolidation;	Improvement
Weerakoon et al,(2022) [28]	0.42	M	CF,PFIC	Lung,Cutaneous	GEN,MER,RIF,EMB,CAM,AZM,AMK, LVFX	5 months	X-RAY:Hyperinflated lungs, peribronchial wall thickening and bilateral lower lobe consolidations; CT:Bilateral cystic bronchiectasis and nodules	Failure
Xuereb et al, (2022) [8]	8	F	CF	Lung	AMK,MER,CFX, AZM,TOB	3 weeks of intravenous antibiotics and three therapeutic bronchoscopies	X-RAY: The collapse of the left upper lobe	Improvement

*CAM* Clarithromycin, *AMK* Amikacin, *AZM* Azithromycin, *CLOF* Clofazimine, *EMB* Ethambutol, *IPM* Imipenem, *LZD* Linezolid, *TGC* Tigecycline, *EMB* Ethambutol, *IMP/CS* Imlpenem/cilastatin; *CFX* Cefoxitin, *SCF* Cefoperazone-sulbactam, *MCFG* Micafungin, *MER* Meropenem, *TOB* Tobramycin, *GEN* Gentamicin, *RIF* Rifampicin, *INH* Isoniazid, *PZA* Pyrazinamide, *LVFX* Levofloxacin, *MOC* Minocycline, *MXF* Moxifloxacin, *CZ* Cefprozil, *AMX* Amoxycillin, *CLI* Clindamycin, *TZP* Piperacilllin-tazobactam, *DOX* Doxycycline, *CIP* Ciprofloxacin, *CVA* Clavulanate; *TZD* Tedizolid, *RMS* Rhabdomyosarcoma, *EP* Ependymoma, *RB* Retinoblastoma, *WT* Wilm’s tumor, *RLL* Right lower lobe, *CHD* Congenital heart disease, *HCE* Hypoxicis chemic encephalopathy, *CCHD* Congenital cyanotic heart disease, *CF* Cystic Fibrosis, *CT* Contrast-enhanced computed tomography, *X-RAY* Chest radiography

*M. abscessus* infection is difficult to diagnose in children. In clinical practice, NTM infection should be identified first, and then the species of pathogen should be further determined from clinical specimens. Currently, mycobacteria are often identified through a combination of pathogen culture and biochemical test technology in clinical practice [32]. Considering some reports of false-positive results from pathogen culture [33], an alternative to traditional laboratory microbial culture combined with biochemical test technology is difficult to identify [32]. mNGS has rapidly emerged as an advanced technique for pathogen detection that can be performed directly on clinical specimens, which is characterized by high speed, high specificity, and high throughput [34]. It is also less affected by previous antibiotic exposure [35]. With the development of NGS technology, differentiating between colonization and infection has also become possible [36]. NGS technology has been used to detect a variety of pathogens, such as *Streptococcus pneumoniae* and *M. abscessus* [37, 38]. In the reported case, we performed several cultures for bacteria and fungi, which were negative. The patient’s clinical symptoms did not improve within 2 weeks of empirical antibiotic treatment, and she developed a dramatic exacerbation of respiratory symptoms. Finally, we detected *M. abscessus* by mNGS of BALF, which showed 515 sequences with significantly higher sequence numbers, and in combination with the presence of immunodeficiency, clinical symptoms, and pulmonary imaging manifestations, the patient was diagnosed with *M*. *abscessus* pulmonary disease according to the diagnostic criteria of the American Thoracic Society [39]. Treatment for *M. abscessus* was then administered; 1 week after treatment, her clinical symptoms were alleviated; and bacterial and fungal cultures of BALF remained negative.

*M. abscessus* is also a special NTM due to its resistance to several antibiotics, resulting in tremendous challenges in the treatment of *M. abscessus* [40]. At the same time, antibiotics' toxic effects and side effects [41] and increasing treatment costs [42] during long-term treatment lead to drug discontinuation or treatment failure. With underlying pulmonary diseases, the course of antibiotic administration may be prolonged, and most patients with pulmonary infection caused by *M*. *abscessus* undergo treatment for at least 12 months [43]. Combination therapy with at least 3 antibiotics is recommended by the 2020 ATS/ERS/ESCMID/IDSA clinical practice guidelines [44]. In this case report, azithromycin and clarithromycin plus imipenem and cilastatin sodium and linezolid were used successively as anti-infective therapy. The patient had an underlying medical condition of ALL, and during chemotherapy, we treated the pulmonary infections concurrently. Considering the potential pulmonary toxicity of various medications and the risk of lung complications caused by infections, we repeated chest CT scans during the treatment. After six months of treatment, the patient no longer had a fever or cough, and lung auscultation was normal. The chest CT scans showed that the infection had been cured. No drug toxicity or side effects occurred. These findings suggest that patients without underlying pulmonary diseases can undergo treatment for less than 12 months, similar to previous findings [45]. As noted in case reports, balancing between the treatment of the primary disease and infection control creates a dilemma, i.e., the risk of disease recurrence may rise due to early termination of chemotherapy without continuing consolidation therapy, while disseminated NTM infection may occur with the continuation of high-dose chemotherapy [46]. Fortunately, ALL treatment was continued throughout anti-infective therapy, and no further acute deterioration of pulmonary function or spreading of the infection was found. After 2.5 years of treatment, the drugs for the patient’s underlying disease (ALL) were discontinued, and she currently remains in hematologic remission.

## Conclusion

Spontaneous pneumomediastinum and subcutaneous emphysema was our patient's main characteristic of chest CT, which is the first such report in a child, thus enriching knowledge regarding imaging changes in *M. abscessus* pulmonary disease in children. This case report reflects good clinical experience in maintaining the balance between chemotherapy and anti-infective therapy for childhood ALL.

### Supplementary Information

**Additional file 1.**

## Data Availability

Sequencing files were deposited into the NCBI SRA database and can be retrieved at https://www.ncbi.nlm.nih.gov/ with accession number PRJNA882796.

## Abbreviations

ALL: Acute lymphoblastic leukemia
CT: Computed tomography
NTM: Nontuberculous mycobacterium
CCCG-ALL-2015: The Chinese Children’s Cancer Group study ALL-2015
VDLP: Vincristine + daunorubicin + L-asparaginase + prednisone
PO_2_: Pressure of oxygen
PCO_2_: Pressure of carbon dioxide
PaO_2_: Arterial oxygen pressure
FiO_2_: Fraction of inspired oxygen
BALF: Bronchoalveolar lavage fluid
mNGS: Metagenomic next-generation sequencing
GM: Galactomannan
